# Cigarette smoking induces the activation of RIP2/caspase-12/NF-*κ*B axis in oral squamous cell carcinoma

**DOI:** 10.7717/peerj.14330

**Published:** 2022-11-04

**Authors:** Yajie Qian, Wenmei Wang, Deyan Chen, Yanan Zhu, Yong Wang, Xiang Wang

**Affiliations:** 1 Nanjing Stomatological Hospital, Medical School of Nanjing University, Nanjing, China; 2 State Key Laboratory of Analytical Chemistry for Life Science & Jiangsu Key Laboratory of Molecular Medicine, Medical School, Nanjing University, Nanjing, China

**Keywords:** Cigarette smoking, Oral squamous cell carcinoma, RIP2, Caspase-12, NF-κB

## Abstract

Cigarette smoking is one of the major risk factors for the occurrence and progression of oral squamous cell carcinoma (OSCC). Receptor-interacting protein 2 (RIP2) has been involved in mucosal immunity and homeostasis via a positive regulation of nuclear factor *κ*B (NF-*κ*B) transcription factor activity. Caspase-12 can bind to RIP2 and dampen mucosal immunity. However, the roles of RIP2/NF-*κ*B and caspase-12 in OSCC induced by cigarette smoking remain unknown. Herein, we investigated the effects of cigarette smoking on the RIP2/NF-*κ*B and caspase-12 in human OSCC tissues and OSCC cell lines (HSC-3). We first observed that RIP2 mediated NF-*κ*B activation and caspase-12 upregulation in OSCC patients with cigarette smoking and cigarette smoke extract (CSE)-treated HSC-3 cells, respectively. Moreover, we confirmed that the downregulation of RIP2 by siRNA resulted in the reduction of caspase-12 expression and NF-*κ*B activity in the presence of CSE treatment *in vitro*. In summary, our results indicated that cigarette smoking induced the activation of the RIP2/caspase-12/NF-*κ*B axis and it played an important role in the development of OSCC. The RIP2/caspase-12/NF-*κ*B axis could be a target for OSCC prevention and treatment in the future.

## Introduction

Oral squamous cell carcinoma (OSCC) is the fifth most common cancer worldwide and occurs in the mouth, including the tongue, gums, cheeks, palate, and bottom of the mouth (Lin et al., 2020b). The worldwide annual incidence of OSCC is over 300,000 cases, with a mortality rate of 48%. OSCC accounts for 90% of all oral cancers, with the highest incidence being observed in men over 50 years of age. Despite the development of new therapies in the treatment of OSCC, the 5-year survival rate is still below 50% in recent decades (Jiang et al., 2020). Hence, there is a need to explore the pathogenesis and progression of OSCC, which would help to find new therapeutic targets.

Several environmental factors and genetic factors have been reported to involve in the process of OSCC (Zeng et al., 2020). At present, smoking is considered to be one of the key factors contributing to carcinogenesis and the progression of oral malignancy (Chien et al., 2021; Tsou et al., 2021). Therefore, an increased risk of developing OSCC was observed among cigarette smokers. However, the role of cigarette smoking in the molecular pathogenesis of OSCC has rarely been assessed. Recent studies have shown that receptor-interacting protein 2 (RIP2) is involved in the occurrence, progression and metastasis of different types of tumors and serves as a prognostic biomarker and therapeutic target (Li et al., 2021; Li et al., 2020). RIP2 also plays an important role in the regulation of inflammation and cell death processes via nuclear factor *κ*B (NF-*κ*B) signaling pathway (Eng, Wemyss & Pearson, 2021). However, the expression of RIP2/NF-*κ*B signaling pathway in OSCC induced by cigarette smoking has not been elucidated.

Philippe et al., indicated that caspase-12 could bind to RIP2, and dampen mucosal immunity (LeBlanc et al., 2008). Our previous studies also showed that caspase-12 could play a vital role in the regulatory effects of cigarette smoking on human beta defensins expression in oral mucosal epithelial cells (Wang et al., 2014). However, the effect and interaction mechanism between RIP2/NF-*κ*B pathway and caspase-12 in the progression of OSCC induced by cigarette smoking is entirely unknown. Therefore, in our present study, we wanted to examine the expression of RIP2/NF-*κ*B pathway and caspase-12 in OSCC patients with cigarette smoking and cigarette smoke extract (CSE)-treated OSCC cell lines, and to explore the interaction between RIP2/NF-*κ*B and caspase-12.

## Materials & Methods

### Patients and tissue specimens

The OSCC tissue samples were collected in Nanjing Stomatological Hospital, Medical School of Nanjing University, and the Ethics Committee of Nanjing Stomatological Hospital, Medical School of Nanjing University approved our study (IRB Approval Number: 2018NL-008 (KS)). Written informed consent was obtained from all the enrolled patients as requested by the Institutional Ethics Committees. Surgical specimens of 40 patients were diagnosed with OSCC and were collected for our study. Pregnant women, lactating women, patients with systemic diseases and other habits, such as alcohol consumption, were excluded from our study. Cigarette smokers included in our study were OSCC patients who had smoked more than 10 cigarettes per day for at least 10 years, while non-smokers were considered patients who had never smoked among OSCC patients.

Our study included 40 patients with OSCC in the groups of smokers and non-smokers, consisting of 24 men and 16 women, with an age range from 30 to 81 years old (median 58 years). The average number of cigarettes among the group of smokers was 20 pack years. Tissue specimens were either immediately processed for immunohistochemistry or stored at −80 °C for western blotting analysis.

### Western blotting

Tissues were rinsed with chilled PBS twice, homogenized by using a high-efficiency tissue grinder, and then lysed in chilled RIPA buffer supplemented with protease inhibitor. The lysates were incubated on ice for 30 min and centrifuged at 14,000 × g for 10 min at 4 °C. The nuclear fractions were separated using a nuclear and cytoplasmic protein extraction kit (KeyGEN BioTECH, Jiangsu, China) according to the manufacturer’s instructions. Total proteins and nuclear proteins were collected and separated by a 10% gradient gel and electrophoretically transferred to polyvinylidene difluoride membranes. The membrane was shaken and sealed for 1 h in 5% BSA at room temperature. The primary antibodies used in our study included RIP2, caspase-12 and NF-*κ*B p65 (1:1000 dilution; Abcam, Cambridge, UK), GAPDH and histone (1:5000 dilution; Cell Signaling, Danvers, MA, USA). The membrane was incubated with the primary antibodies overnight at 4 °C, followed by each corresponding secondary antibody at room temperature for 1 h at 37 °C. Finally, using ECL kits (Pierce, Rockford, IL, USA), the data of the target protein were first normalized to GAPDH or histone.

### Immunohistochemistry

Serial tissue sections (4 µm thick) were sliced from paraffin-embedded formalin-fixed tissues, and immunohistochemical staining was performed. Briefly, tissue sections were stained using specific primary antibodies, biotin-conjugated secondary antibodies, and horseradish peroxidase (HRP)-conjugated avidin. Subsequently, specific antibody interactions were detected with the HRP substrate 3,3′-diaminobenzidine (DAB). Finally, sections were rinsed and counterstained with hematoxylin. The primary antibodies used were mouse monoclonal anti-RIP2 (1:50 dilution), rabbit polyclonal anti-caspase-12 (1:800 dilution), and rabbit polyclonal anti-NF-*κ*B p65 (1:400 dilution).

Densitometry image analysis was performed using the procedure described in a previous study (Qian et al., 2015). The densitometry analysis of immunohistochemical results was performed by a blinded investigator using Image-Pro Plus analysis software (version 6.0; Dallas, TX, USA). Ten discontinuous fields (400 ×) were randomly selected from each section for evaluation, and the color segmentation algorithm was then used to separate the contributions of the DAB and hematoxylin dyes. The average value of the optical densities of all selected pixels was the mean optical density (integrated option density/area).

### Preparation of CSE

The cigarettes used in the study (3R4F) were manufactured by the Tobacco Research Institute, University of Kentucky (Lexington, KY, USA). CSE was prepared using a protocol similar to that previously described (Giebe et al., 2021; Ye et al., 2021; Zeller et al., 2019). Briefly, 3R4F was lit and suctioned with a miniature desktop vacuum pump to 10 ml of cell growth medium at a speed of 50 ml/min. After filtration with a 0.22-µm pinhead filter and adjusting the pH to 7.45, the filtered aliquot was used as the 100% CSE original solution. Then, the CSE original solution was stored at −20 °C for use in *in vitro* assays, to avoid repeated freeze-thaw.

We measured the contents of CSE after thawing, including nicotine, using an Agilent 1200 series rapid resolution LC system (Agilent Technologies Inc., Santa Clara, CA, USA). The chromatographic separation was achieved on an Ultimate XB-C18 column, 150  × 4.6 mm i.d. with 5-µm particles (Welch Materials, Shanghai, China). The separation was performed at a flow rate of 1.0 ml/min, and the column temperature was 25 °C. The quantitation of nicotine was performed using calibration curves of peak area versus nicotine concentration. The concentration of nicotine within 100% CSE ranged from 1.65 to 1.86 µg /ml.

### Cell culture

The OSCC cell line (HSC-3) was purchased from ATCC and used in our study. The cell culture medium contained Dulbecco’s modified Eagle medium (DMEM) (Gibco, Grand Island, NY, USA), penicillin (100 U/L), streptomycin (100 U/L) and 10% (v/v) fetal bovine serum (Gibco). Fresh medium was supplemented every 2–3 days. Cells were then treated with different concentrations of CSE (0, 0.5%, 1%, 2%, 4%) for 24 h in a 37 °C constant temperature incubator containing 5% CO_2_.

### siRNA interference

To inhibit RIP2 expression, small interfering RNA (siRNA) against RIP2 was used. The RIP2 siRNA (∼50 nM in each well of 6-well-plate) was added to Opti-MEM reduced serum medium at a concentration of 100 pmol/µl, mixed with 5 µl of Lipofectamine 3000, and maintained at room temperature for 20 min. For the transfection assay, 5 × 10^6^ cells were seeded in 6-well plates and incubated overnight at 37 °C in 5% CO_2_ until 60%–80% confluence was achieved and were then transfected with siRNA. RIP2 siRNA sense: CACCAATCCTTTGCAGATAAT.

### Statistical analysis

Statistical analyses were performed using SPSS 22.0 statistical software (IBM Corporation, USA) and GraphPad Prism 6.0 (La Jolla, CA, USA). All continuous data are reported as the mean ± SD. Data normality tests were carried out by the Shapiro - Wilk statistical test. The student *t*-test and One-way ANOVA were used for normally distributed data, and comparisons between each group were performed using *Dunnett’s* test. *P* < 0.05 was considered statistically significant.

## Results

### Cigarette smoking induced RIP2-mediated NF-*κ*B activation in OSCC patients

**Figure 1:**
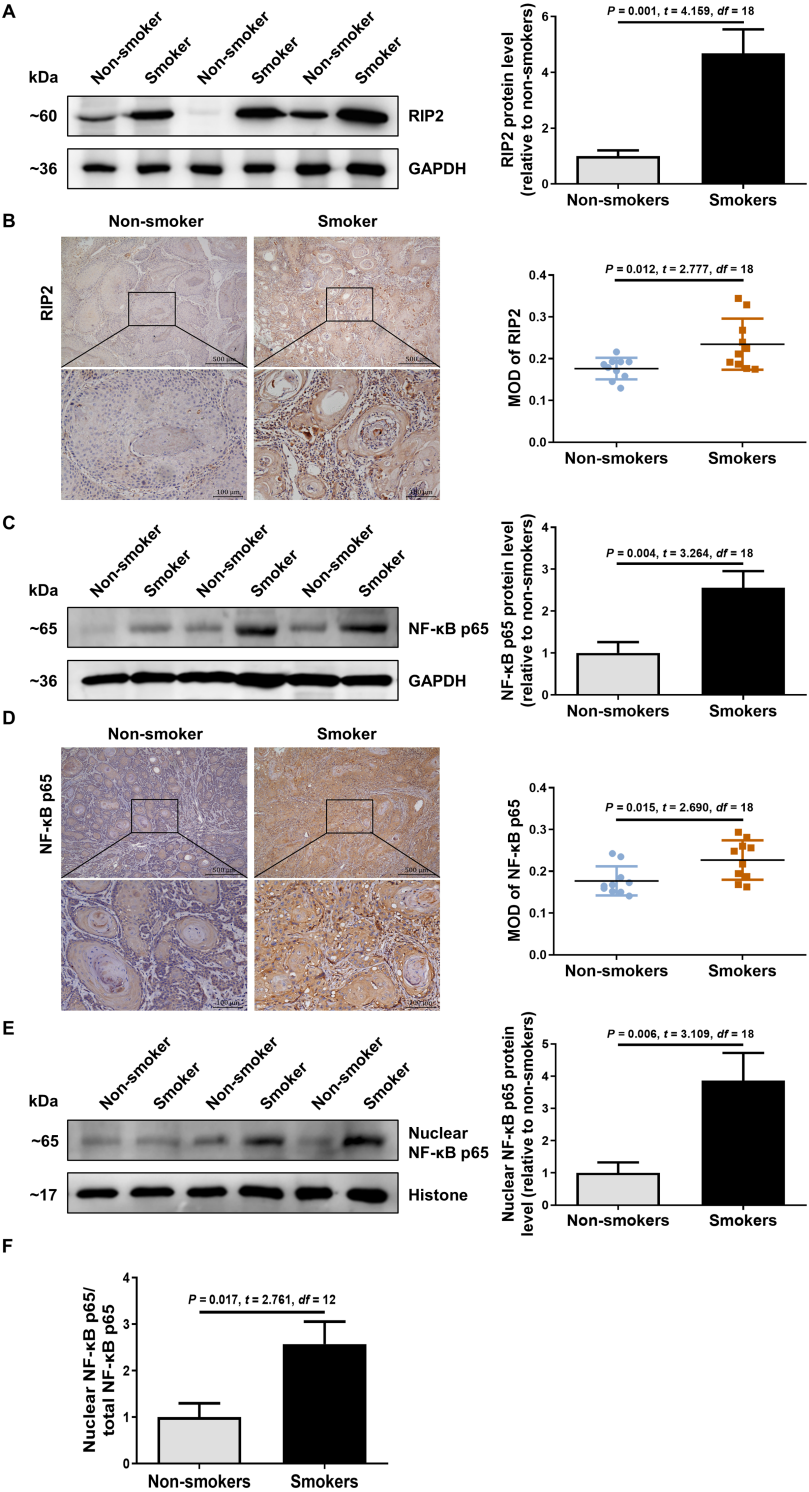
Cigarette smoking activated RIP2/NF-*κ*B pathway in patients with OSCC. (A) Western blotting analysis was performed to assess RIP2 protein level in the oral mucosa tissues of OSCC patients between non-smokers and smokers. The RIP2 protein of the mean expression in the group of smokers was first normalized to GAPDH and then they were as compared to the group of non-smokers using the student *t-* test. *n* = 10. (B and D) Representative immunohistochemical staining of RIP2 (B) and NF-*κ*B p65 (D) in the oral mucosal epithelium of non-smokers and smokers. Scale bars: 500 µm. MOD, mean optical density. (C and E) Total NF-*κ*B p65 protein and nuclear NF-*κ*B p65 protein were evaluated by western blot assay, normalized to GAPDH and Histone in the oral mucosal epithelium of non-smokers and smokers, respectively. *n* = 10. (F) The histogram showed the corresponding quantification of nuclear NF-*κ*B p65, relative to total NF-*κ*B p65.

To explore the possible role of RIP2/NF-*κ*B in OSCC patients with cigarette smoking, we first detected the levels of RIP2 and NF-*κ*B expression by using western blotting and immunohistochemical assays. Interestingly, compared with the non-smoker group, RIP2 was markedly increased in the smoker group (Fig. 1A). Immunohistochemistry analysis showed that RIP2 staining was negative in non-smokers, and stronger in the OSCC tissues of smokers. The positive expression of RIP2 was mainly located in the cytoplasm of tumor cells (Fig. 1B). Consistently, results also indicated that NF-*κ*B p65 expression was increased in the OSCC tissues of the smokers compared with those of the non-smokers (Fig. 1C). Moreover, we observed positive staining for NF-*κ*B p65 in the cytoplasm and nucleus of tumor cells in the OSCC tissues of smokers (Fig. 1D). As NF- *κ*B is a nucleus-cytoplasm shuttling protein, we further analyzed whether its cellular localization changes after stimulation with cigarette smoking. The results indicated that cigarette smoking promoted nuclear translocation of NF-*κ*B p65 in OSCC tissues (Figs. 1E and 1F). Together, these data indicated that cigarette smoking induced RIP2-mediated NF- *κ*B activation in the OSCC tissues of smokers.

### RIP2 mediated NF-*κ*B activation correlated with the upregulation of caspase-12

**Figure 2:**
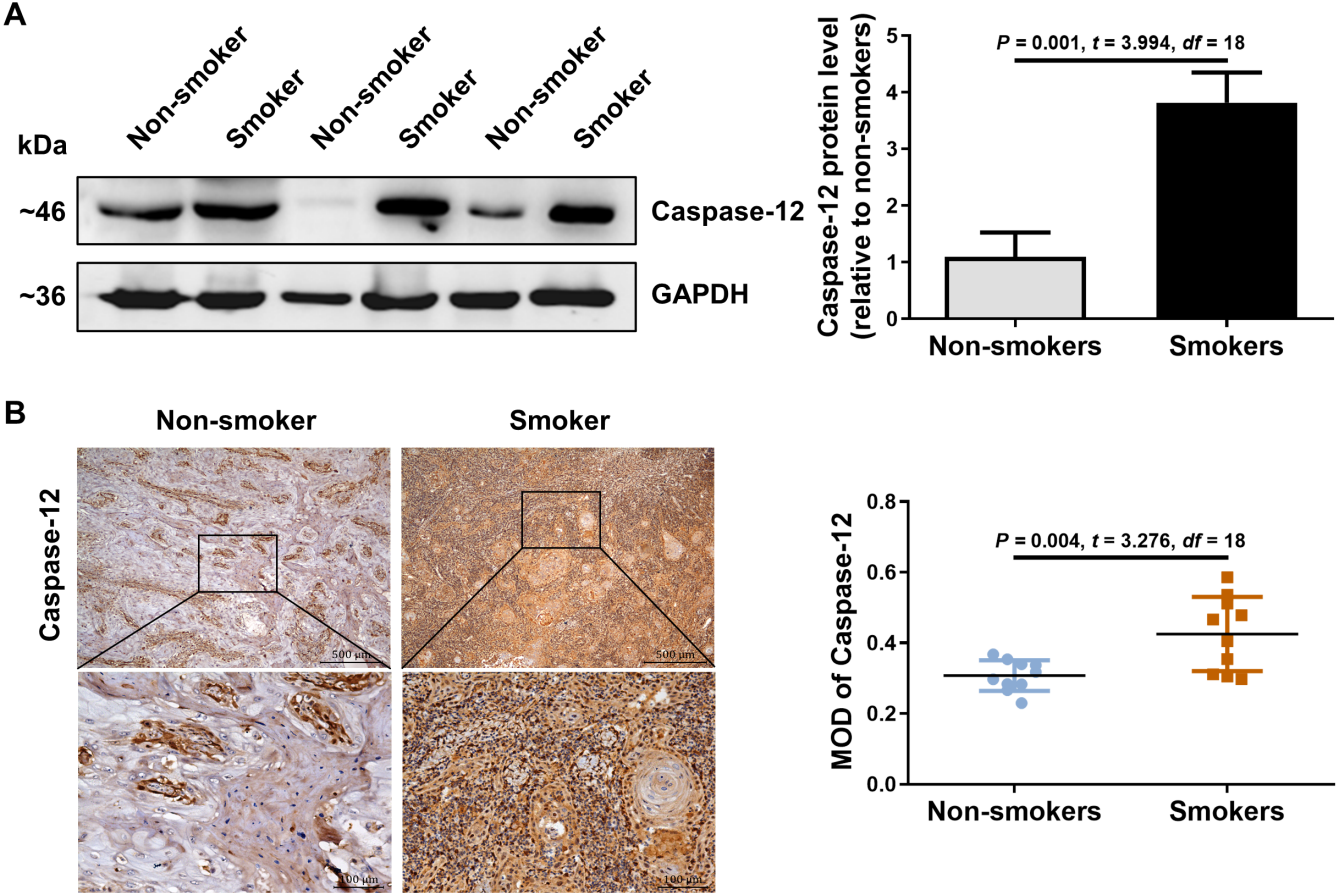
Caspase-12 increased in smokers compared with non-smokers among oral mucosa of OSCC patients. (A and B) Caspase-12 protein was analyzed by western blotting and immunohistochemical assays, respectively. The expression of caspase-12 in the group of smokers was normalized to GAPDH and then were compared to the group of non-smokers using the student *t-* test. *n* = 10. Scale bars: 500 µm. MOD, mean optical density.

We further detected the expression of caspase-12 in the OSCC tissues of smokers. As shown in Fig. 2A, caspase-12 protein was obviously higher in the OSCC tissues of smokers than in those of non-smokers. Moreover, immunohistochemical assays also revealed that a significantly stronger staining intensity of caspase-12 in the tumor cells and stroma was observed in the OSCC tissues of smokers than in those of non-smokers (Fig. 2B). The above results indicated that the expression of RIP2/NF-*κ*B was positively correlated with that of caspase-12 in OSCC tissues.

### CSE promoted activation of RIP2/NF-*κ*B and upregulated caspase-12 in HSC-3 cells

**Figure 3:**
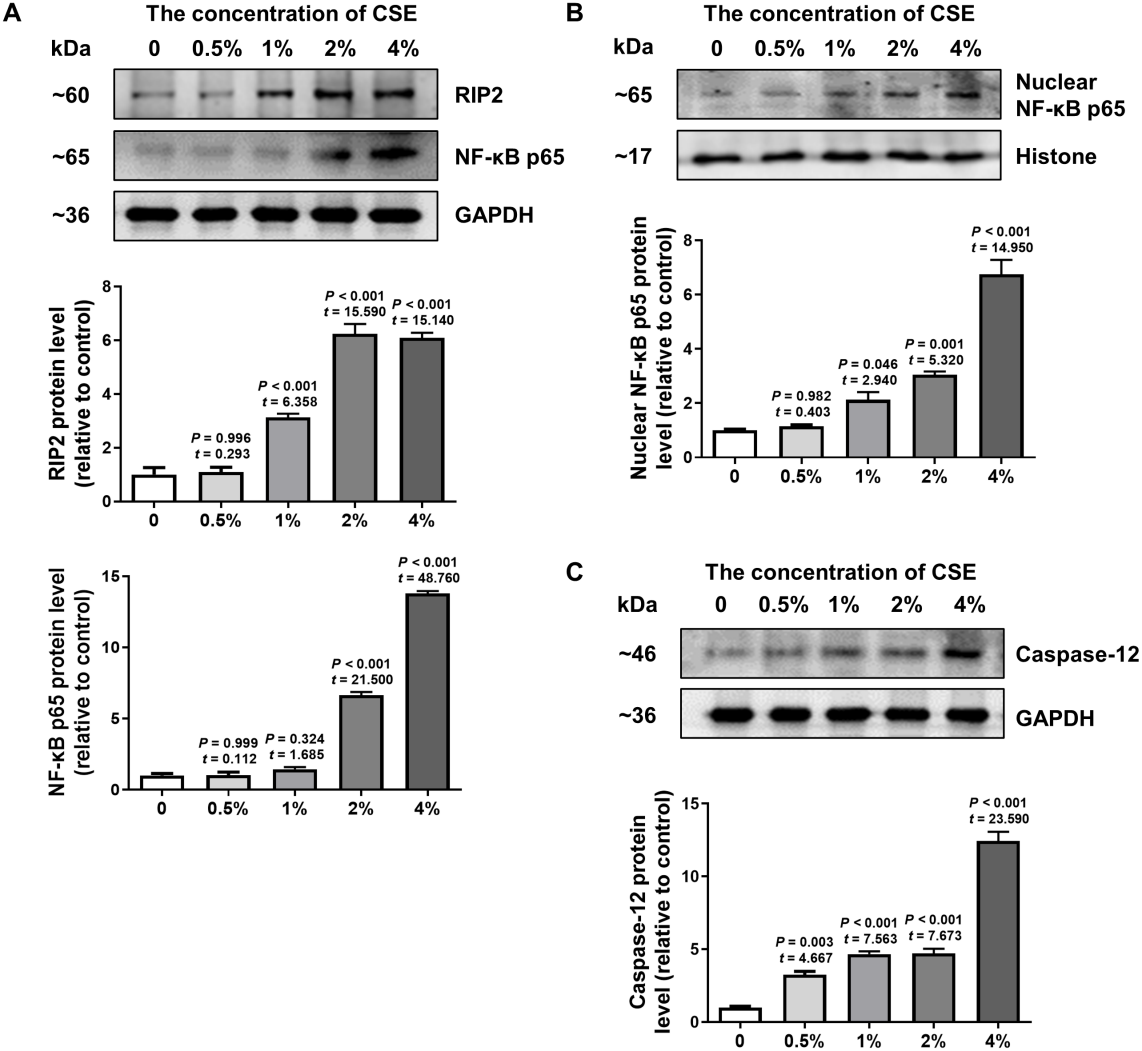
Different concentrations of CSE treatment promoted RIP2/NF-*κ*B and caspase-12 protein in HSC-3 cells. Western blotting was used to analyze the levels of total RIP2, NF-*κ*B p65 (A), nuclear NF-*κ*B p65 (B), and caspase-12 (C) in HSC-3 cells in the presence of different concentrations of CSE. The target proteins of the mean expression in the CSE-treated HSC-3 cells were first normalized to GAPDH and then were compared to the group of non-treated HSC-3 cells. The One-way ANOVA was used for normally distributed data, and comparisons between each group were using Dunnett’s test. *df* = 10.

To further confirm the effect of cigarette smoking on the RIP2/NF-*κ*B signaling pathway and the expression of caspase-12, we examined the levels of RIP2, NF-*κ*B and caspase-12 expression in HSC-3 cells by using western blotting. The results in Fig. 3A indicated that CSE induced activation of the RIP2/NF-*κ*B pathway in a dose-dependent manner (Fig. 3A). To further identify the effect of CSE on NF-*κ*B-mediated transcriptional activity, the nuclear protein NF-*κ*B p65 in HSC-3 cells was measured. Our results clearly showed that CSE treatment induced the activation of the NF-*κ*B signaling pathway (Fig. 3B). In addition, the protein level of caspase-12 was obviously increased after stimulation with CSE (Fig. 3C). Our *in vitro* results were similar to the results obtained in OSCC tissues. These results indicated that the RIP2/NF-*κ*B pathway was activated and caspase-12 expression was increased after CSE treatment.

### RIP2 positively regulated caspase-12 expression in CSE-treated HSC-3 cells

**Figure 4:**
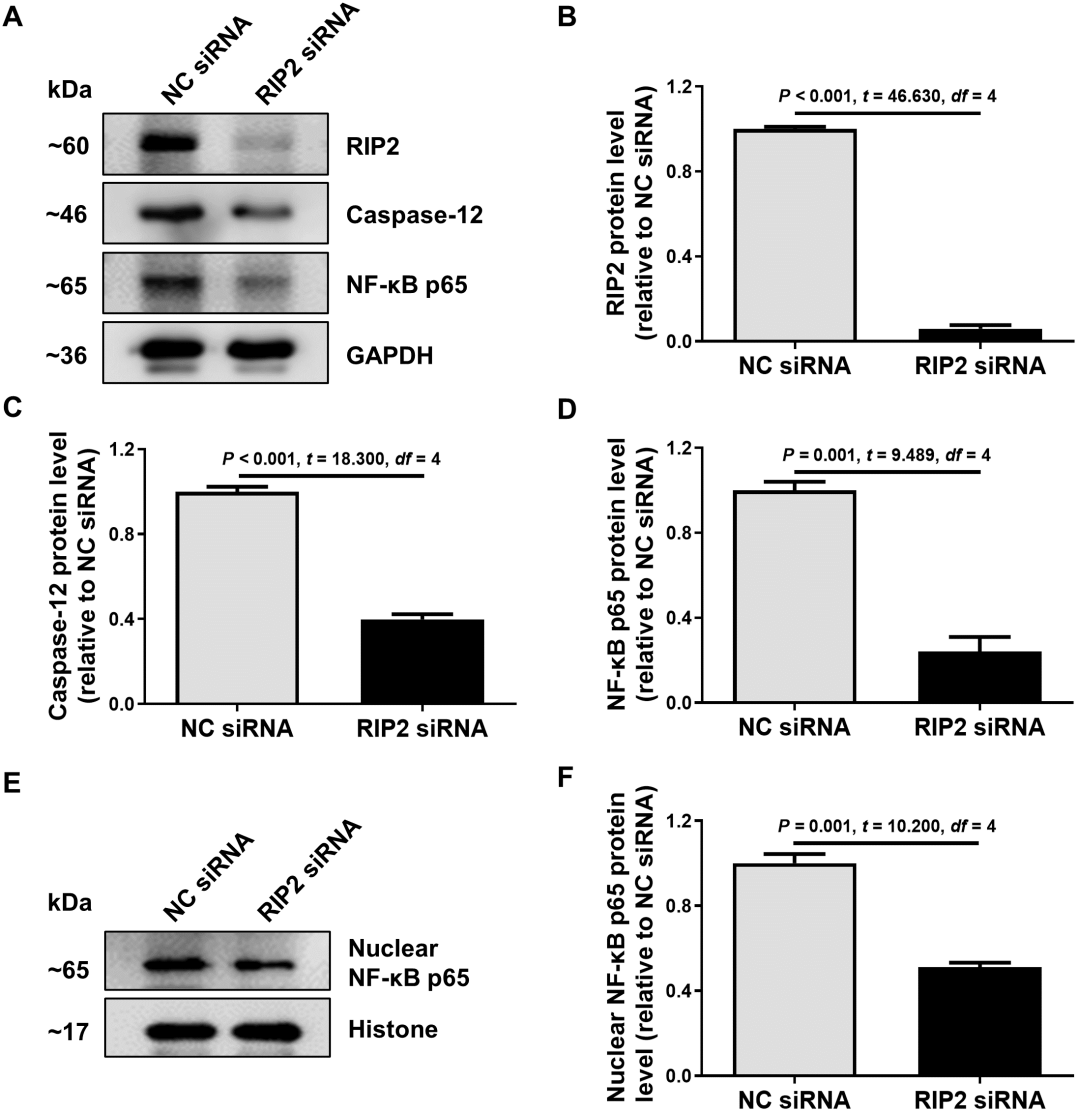
RIP2 knockdown mediated the downregulation of caspase-12 and NF-*κ*B in CSE-treatment HSC-3 cells. (A) The levels of total proteins, including RIP2, caspase-12, and NF-*κ*B p65, were analyzed by western blotting in HSC-3 cells transfected with RIP2 siRNA or NC siRNA for 24 h before treatment with 4% CSE. (B–D) The results were normalized to GAPDH, and compared using the student *t-* test. (E) The expression level of NF-*κ*B p65 in the nucleus was further detected by western blotting analysis. The results were normalized to histone expression, and compared using the student *t-* test (F).

To further confirm the relationship between RIP2 and caspase-12 in the presence of CSE treatment, we used siRNA to reduce the expression of RIP2 in HSC-3 cells. We found that the knockdown of RIP2 significantly reduced the levels of caspase-12 and total NF- *κ*B p65 expression in CSE-treated HSC-3 cells (Figs. 4A–4D). The effect of RIP2 knockdown also resulted in a reduction in the accumulation of nuclear NF-*κ*B p65 after treatment with CSE (Figs. 4E and 4F). These results indicated that RIP2 positively regulates the NF-*κ*B pathway and caspase-12 expression, which may be involved in the development of OSCC induced by cigarette smoking.

## Discussion

In recent years, there has been an increase in the incidence of OSCC, according to growing evidence (Zang et al., 2022). It adversely affects the appearance and quality of life in OSCC patients. OSCC continues to have a poor prognosis despite significant advances in therapeutic intervention. Therefore, there is an urgent need to find potential mechanisms and therapeutic targets for OSCC.

RIP2 involves in the intracellular innate immune response and participates in multichannel signal transduction (Gong et al., 2018; Lin et al., 2020a). Moreover, the dysregulation of RIP2 signaling pathway ultimately promotes the development of cancers (Zare et al., 2018; Zhou et al., 2021). Chan et al. (2016) showed that higher expression of RIP2 protein presented in head and neck squamous cell carcinoma tissue than in noncancerous matched tissue. Our results indicated that RIP2 expression obviously increased in the OSCC patients with cigarette smoking and CSE-treated HSC-3 cells. RIP2 activation induced by CSE treatment may drives proinflammatory and exhibit antimicrobial responses in human oral mucosal epithelial cells. Our present findings are consistent with a previous study that cigarette smoking upregulated RIP2 expression in lung tissues in mice and silencing RIP2 gene could ameliorate acute lung injury (Dong et al., 2019). The above evidence suggested that an increase of RIP2 protein could directly correlate with cigarette smoking involving in the progression of OSCC.

After RIP2 activation, NF-*κ*B dimers (such as p65/p50) translocases into the nucleus and triggers the transcription of target genes (Hoesel & Schmid, 2013). The activation of NF-*κ*B further promotes the proliferation of tumor cells and accelerates angiogenesis and contributes to the initiation and progression of different kinds of tumors (Inoue et al., 2018; Yu et al., 2020). Therefore, NF-*κ*B pathway is a promising therapeutic target in the clinical practice (Hyder et al., 2020). In our study, we found that the expression of NF-*κ*B was activated in both smokers with OSCC and CSE-treated HSC-3 cells. Studies have shown that NF-*κ*B is an important molecule in response to cigarette smoke (Le et al., 2020; Zhang et al., 2016). CSE strengthened the transcriptional activity of NF-*κ*B *via* promoting its nuclear translocation and DNA binding activity in human A549 cells(Qiu et al., 2017). On the contrary, other studies showed that cigarette smoke did not increase the activation of NF-kB in the lung (Tharappel et al., 2010) or human umbilical vein endothelial cells (Chen et al., 2009). Inconsistent conclusions of cigarette smoke on NF-*κ*B expression among different studies may depend on different cell types. When we tried to downregulate the expression of RIP2, we observed that the translocation of NF-*κ*B p65 in the nucleus could be hampered. These results indicate that CSE triggers the translocation of NF-*κ*B p65 into the nucleus in a RIP2-dependent manner. Similarly, one study showed that silencing of RIP2 suppressed the growth of gastric cancer cell growth by inhibiting cell migration and inducing apoptosis through the NF-*κ*B signaling pathway (Yang et al., 2021).

Caspase-12 is a cysteine protease and plays an important role in innate immune responses and inflammation, as well as in cell death (Zhang et al., 2021). Furthermore, recent studies have also confirmed that the stimulation of cigarette smoke could activate the expression of caspase-12 (Ding et al., 2018; Tan et al., 2017). Our results showed that caspase-12 was increased among OSCC patients with cigarette smoking and CSE-treated HSC-3 cells, respectively. Therefore, the available evidence supports that cigarette smoking upregulates caspase-12 in OSCC. In addition, the downregulation of caspase-12 induced by RIP2 siRNA in CSE-treated HSC-3 cells supported that caspase-12 may be a downstream molecular of RIP2 in cigarette smoking-induced OSCC. Recent studies have revealed that excessive caspase-12 mediated I *κ*B *α* degradation and significantly increased the activation of NF-*κ*B activity (Chow et al., 2021; Chu et al., 2017). Accordingly, we hypothesize that smoking promotes caspase-12 interaction with RIP2 and further activates NF-*κ*B participating in the progression of OSCC.

## Conclusions

In summary, our data suggested that cigarette smoking could upregulate RIP2/caspase-12/NF-*κ*B in OSCC. From a mechanistic perspective, the increased expression of RIP2 was essential for the activation of caspase-12 and could activate NF- *κ*B. In conclusion, our results indicated that cigarette smoking may promote the progression of OSCC through the RIP2/NF- *κ*B signaling pathway, and this process is closely related to caspase-12. RIP2 may be a viable pharmacological target to modulate the development of OSCC. Further studies are needed to determine the specific mechanism underlying the activation of the RIP2/caspase-12/NF-*κ*B axis in OSCC due to cigarette smoking.

##  Supplemental Information

10.7717/peerj.14330/supp-1 Supplemental Information 1 Raw numeric data

10.7717/peerj.14330/supp-2 Supplemental Information 2 Uncropped western blots

10.7717/peerj.14330/supp-3 Supplemental Information 3 Cigarette smoking induces the activation of RIP2/caspase-12/NF-*κ*B axis in OSCC

## Additional Information and Declarations

### Data Availability

The following information was supplied regarding data availability: The raw measurements are available in the Supplementary File.
