# Supplementary figures and images for: Cigarette smoking induces the activation of RIP2/caspase-12/NF-κB axis in oral squamous cell carcinoma
Source: PeerJ. 2022 Nov 4;10:e14330. doi: 10.7717/peerj.14330 (PMC9639427; doi:10.7717/peerj.14330)

Fig. 1


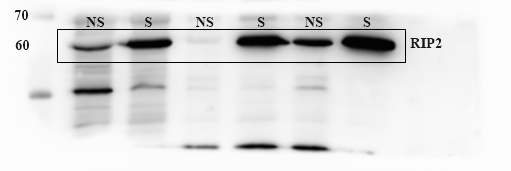

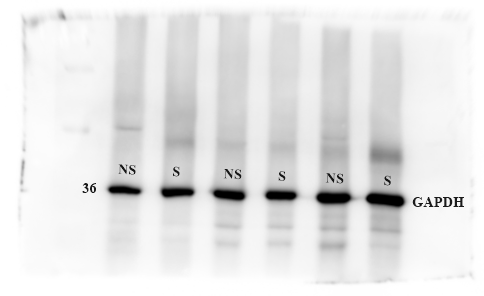


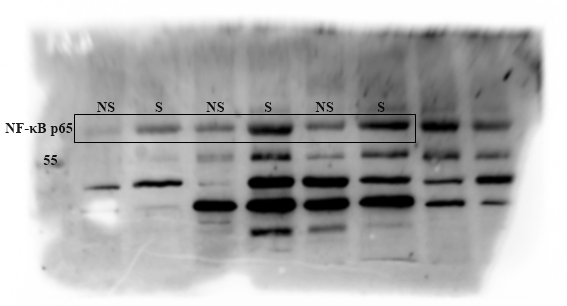

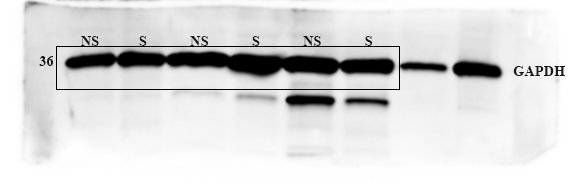


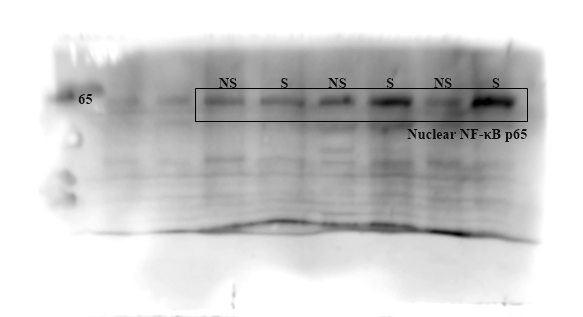

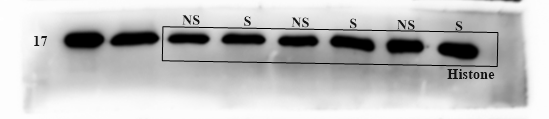


Fig. 2


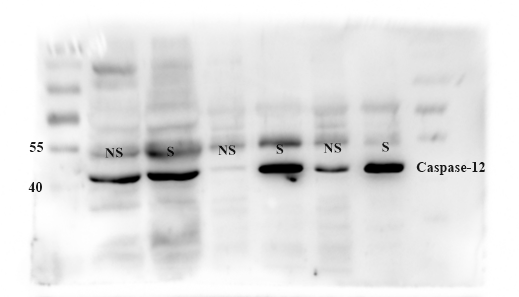




Fig. 3


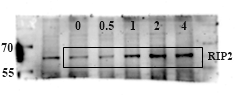

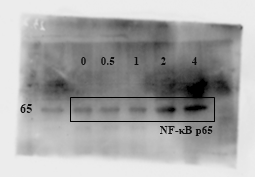

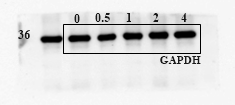


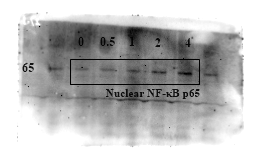

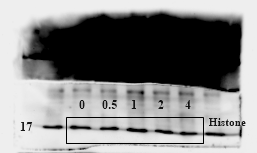


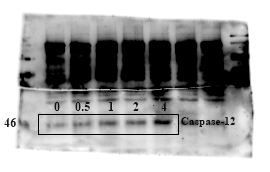

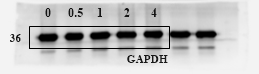


Fig. 4


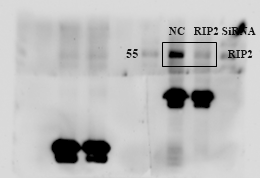

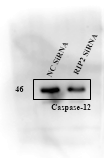


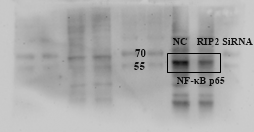

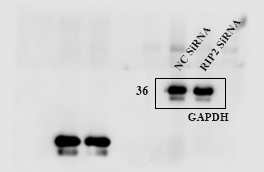


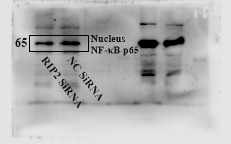

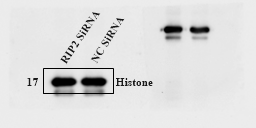

Supplement: Supplemental Information 2 [file peerj-10-14330-s002.docx]

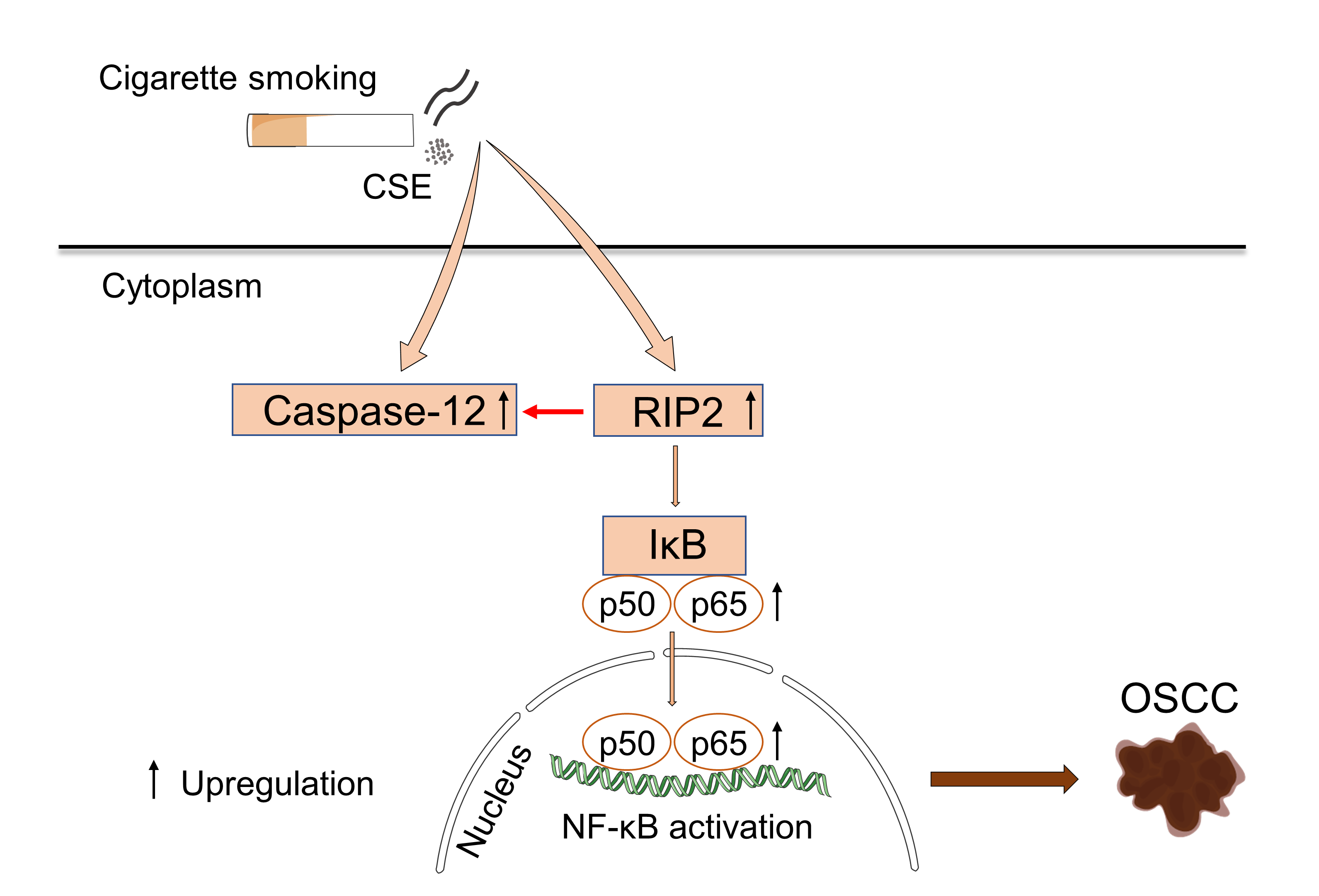

Supplement: Supplemental Information 3 [file peerj-10-14330-s003.png]
